# Effect of Early‐Onset Dementia on Job Loss in Japan: A Matched Cohort Database Study Using Health Insurance Claims Data

**DOI:** 10.1111/psyg.70117

**Published:** 2025-11-28

**Authors:** Kenta Sumitomo, Yoichi Higashibeppu, Masataka Tajima, Hitoshi Sato, Erika Sugiyama

**Affiliations:** ^1^ Department of Pharmacokinetics and Pharmacodynamics Showa Medical University Graduate School of Pharmacy Tokyo Japan; ^2^ Eisai Co. Ltd. Tokyo Japan; ^3^ Faculty of Pharmacy Mahidol University Bangkok Thailand

**Keywords:** database study, early‐onset dementia, job loss

## Abstract

**Objective:**

To assess the rate of job loss amongst primary insured participants diagnosed with early‐onset dementia (EOD) and to investigate the association between patients' background factors and job loss. Additionally, we evaluated the job loss rates amongst primary insured participants whose spouses were diagnosed with EOD.

**Methods:**

We analysed health insurance claims data from the Japan Medical Data Centre, covering the period from 1 April 2013, to 30 November 2023. Participants were categorised into two groups: EOD Group 1, comprising primary insured individuals with EOD; EOD Group 2, comprising primary insured individuals' spouses with EOD. Each group was compared with its respective control group. The control group comprised participants without dementia matched in a ratio of 1:5 to cases in EOD group based on sex, age and major comorbidities.

**Results:**

The rate of job loss in EOD Group 1 was higher than that in Control Group 1 (hazard ratio [HR] = 1.59, 95% confidence interval [CI]:1.39–1.82, *p* < 0.05). In contrast, EOD Group 2 had a lower rate of job loss than Control Group 2 (HR = 0.75, 95% CI: 0.57–0.996, *p* = 0.046). In EOD Group 1, patient background factors influencing job loss within 2 years included sex (female) (adjusted HR = 2.18, 95% CI:1.60–2.96, *p* < 0.05), age (adjusted HR = 1.07, 95% CI: 1.04–1.10, *p* < 0.05) and hypertension (adjusted HR = 1.35, 95% CI: 1.02–1.77, *p* < 0.05).

**Conclusion:**

This study identified participant background factors that influence job loss amongst participants with EOD, suggesting that addressing comorbidities at EOD onset and promoting lifestyle modifications may be beneficial. Examining the time to job loss following EOD onset is crucial for predicting its economic impact and developing effective support strategies. Therefore, this study's findings may be valuable in reducing the rate of job loss by enforcing workplace health promotions for employees with EOD.

## 1 Introduction

In Japan, approximately 4.62 million people had dementia in 2012, accounting for 15% of the older population aged ≥ 65 years [1]. Projections indicate that the prevalence of dementia is expected to reach 20% by 2030 and 25% by 2045 [2]. Of the patients with dementia, those with early‐onset dementia (EOD), defined as dementia occurring under 65 years of age, are estimated to be 35 700, and the national EOD prevalence rate is estimated to be 50.9 per 100 000 population. The most common type of EOD is Alzheimer's disease (52.6%), followed by vascular dementia (17.1%) and frontotemporal dementia (9.4%) [3].

Notably, some patients with EOD are employed and may have young children who are not financially independent at the time of the disease onset. Consequently, the impact of EOD on patients and their families is more profound than that of dementia diagnosed at ≥ 65 years. Furthermore, some patients with EOD are forced to leave their jobs, disrupting their economic stability [4, 5, 6]. The onset of EOD in the patient may lead to the discontinuation of employment. Alternatively, if the spouse develops EOD, the primary insured individual may be obliged to withdraw from the workforce due to caregiving responsibilities. In either circumstance, there is a marked decline in household income, potentially exerting adverse effects on the overall economic environment. In addition, their spouses and children may have to assume a caregiving role, and the patients and their spouses may experience psychological stress and social isolation [7, 8]. Therefore, understanding the duration until job loss following EOD onset in the patients themselves as well as the duration until job loss amongst primary insured individuals whose spouses develop EOD, is essential. Such information is crucial for predicting the future economic environment and developing appropriate countermeasures, such as utilising national support systems. In addition, examining whether patient background factors such as comorbidities at the time of EOD diagnosis influence job loss could provide valuable insights. If comorbidities affect employment duration, targeted treatment at the time of EOD onset may prolong job retention. Furthermore, knowing the approximate length of time until job loss enables companies to prepare for employee replacement. Therefore, this information is important to patients and society.

Research on EOD in Japan is currently limited and is mainly based on questionnaire surveys. Questionnaire‐based studies are well suited for assessing patient status at a specific point in time, such as determining prevalence rates and subtype distributions. However, when studying diseases with a limited number of cases, questionnaires have to be distributed to numerous institutions, and the response rates are often low, making the process labor‐intensive. Given these challenges, a database study may be more suitable than a questionnaire‐based study for studies that require a long time to examine the period until job loss rather than a single point in time.

In addition, research on job loss amongst patients with EOD is limited worldwide. A previous study reported the time to job loss [9]; however, it only included patients up to 59 years, excluding those aged ≥ 60 years diagnosed with EOD. Furthermore, the relationship between patient background factors such as comorbidities and job loss was not examined.

Therefore, we aimed to evaluate the job loss status of patients diagnosed with EOD up to the age of 64 years over a 2‐year period and to investigate the relationship between job loss and patient background factors using a database analysis—a relationship that had not been examined in previous database studies. We further enable a more rigorous comparison than the previous study by constructing a matched control group using patient factors, including comorbidities. Additionally, the rate of job loss amongst primary insured participants whose spouses are diagnosed with EOD was also examined.

In addition, considering that dementia is a progressive and irreversible neurodegenerative disease with a long disease duration, long‐term information on job loss amongst patients and their spouses is essential. Therefore, we also monitored job loss status for up to 7 years, a longer follow‐up period than in previous studies, after securing a sufficient number of cases.

## 2 Methods

### 2.1 Data Source

We conducted a matched cohort analysis using health insurance claims data provided by the Japan Medical Data Centre Inc. [10, 11]. This database is unique to Japan and comprises anonymised and standardised inpatient, outpatient and pharmacy claims for employees and their family members from large occupation‐based health societies. In Japan, all citizens are required to enrol in some form of public health insurance system, through which a portion of medical expenses is covered by insurance, and detailed healthcare utilisation data are accumulated via insurance claims [12, 13]. The database contains detailed information, including sex, age, diagnoses coded with the International Classification of Diseases, 10th Edition (ICD‐10), comorbidities, prescribed drugs and duration of prescriptions. Based on this system, the JMDC database primarily included employees of large companies and their family members enrolled in health insurance societies. As of Feb 2025, the total number of registered participants is approximately 21 million. This study utilised data from 1 April 2013 to 30 November 2023.

### 2.2 Study Population

We categorised the participants into four groups: (1) EOD Group 1, comprising primary insured participants diagnosed with EOD; (2) Control Group 1, comprising primary insured participants without dementia; (3) EOD Group 2, comprising primary insured participants' spouses with EOD and (4) Control Group 2 comprising primary insured participants' spouses without dementia. We investigated the duration until job loss amongst primary insured participants in each group. EOD Group 1 comprised primary insured participants aged 40–64 who had received their first dementia diagnosis and had a record of prescription for anti‐dementia drugs, such as donepezil, galantamine, rivastigmine and memantine. Dementia was defined based on ICD‐10 criteria (F00–03 and G30–G31, except for G319) [9]. The index date was defined as the date of first diagnosis of dementia or the date of the first prescription of the anti‐dementia drug, whichever occurred earlier. We included only cases that had been enrolled in the health insurance system for at least 365 days before the index date. Control Group 1 comprised participants without dementia matched to cases in EOD Group 1 based on sex, age and major comorbidities, with a matching ratio of 1:5. Compared to one‐to‐one matching, adopting a one‐to‐many matching approach offers several advantages, including improved estimation precision, more efficient use of available data and the ability to incorporate a larger pool of control participants. These benefits are expected to enhance the overall validity of the estimates. Furthermore, a 1:5 matching ratio was selected to ensure consistency with previous relevant studies and to maintain methodological comparability. The selected major comorbidities included hypertension [14, 15, 16, 17], hyperlipidaemia [18, 19], diabetes [15, 20, 21, 22], cerebral infarction [23, 24] and depression [25, 26, 27], all of which have been reported to be potentially associated with cognitive decline and the development of dementia. The definition of each comorbidity is provided in Table S1.

EOD Group 2 comprised primary insured participants' spouses aged 40–64 diagnosed with dementia for the first time and had a record of a prescription for anti‐dementia drugs under conditions similar to those applied to EOD Group 1. Control Group 2 comprised primary insured participants' spouses without dementia, matched to cases in EOD Group 2 using criteria similar to those used for Control Group 1.

### 2.3 Outcome Measures

We defined withdrawal from health insurance as job loss, as done in a previous study [9] and investigated the period from EOD onset to job loss for comparison with the prior research. For EOD Group 1, the period was defined as the time from EOD onset amongst the primary insured participant until their job loss. In EOD Group 2, the period was defined as the time from EOD onset in the primary insured participant's spouse to the job loss of the primary insured participant. In addition, the relationship between patient backgrounds, such as sex, age and comorbidities, and the duration until job loss was examined for EOD Group 1.

### 2.4 Statistical Analysis

Summary statistics were calculated for participant background variables, including sex, age at dementia diagnosis and comorbidities at the index date, across all four groups. Baseline characteristics were summarised using descriptive statistics. Kaplan–Meier curves were constructed to calculate the cumulative incidence rates of job loss and their 95% confidence intervals (CIs) at 2 and 7 years after the index date. Hazard ratios (HRs) were estimated using stratified Cox proportional hazard (PH) models. Statistical significance was defined as a two‐sided *p* < 0.05. The HR and 95% CI for the variable value were calculated using the Cox PHs model. HR was computed by exponentiating the coefficient *β* (HR = exp(*β*)), and CI was estimated as exp(*β*‐1.96·SE), exp(*β*+1.96·SE) based on the standard error (SE). Significant results are highlighted when the CI exceeds or falls below 1. We assessed the PHs assumption prior to fitting the Cox PHs model. Kaplan–Meier curves and log(−log) survival plots were visually inspected for major crossings. Martingale residuals were used to assess the linearity of continuous covariates. We further applied Schoenfeld residual tests (cox.zph function, survival package in R) to formally examine time‐dependent effects. No significant violation of the PH assumption was observed. Statistical analyses were performed using R software, version 4.4.1.

## 3 Results

**FIGURE 1:**
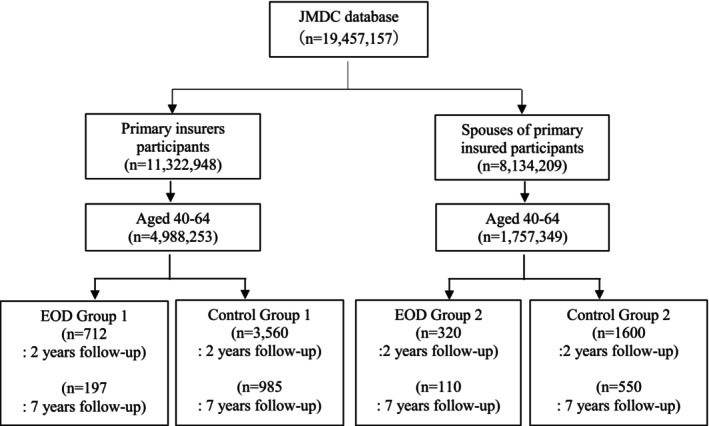
Flow diagram of included participants.

The database includes data from 19 457 157 participants. Of these, 4 988 253 primary insured participants aged 40–64 years had at least 2 years of observation data. From this subset, 712 participants who developed EOD were classified into EOD Group 1. Matching for EOD Group 1 was conducted based on sex, age and major comorbidities related to incident dementia, resulting in Control Group 1, which comprised 3560 participants (Figure 1). Conversely, the database included 8 134 209 spouses of primary insured participants, 1 757 349 of whom were aged between 40 and 64 years and had at least 2 years of data available. From this subset, 320 primary insured participants' spouses who developed EOD were classified into EOD Group 2. Matching for EOD Group 2 was conducted based on similar criteria, resulting in Control Group 2, which comprised 1600 participants.

Tables 1 and S2 show the background characteristics of the participants in the four groups at the index data, with follow‐up periods of 2 and 7 years, respectively. The proportion of women in EOD Groups 1 and 2 was 13.6% and 95.6%, respectively. This indicates that the majority of primary insured participants who developed EOD were men, and the majority of primary insured participants' spouses who developed EOD were women. EOD onset was most frequent in the 60–64 age group (EOD Group 1: 39.7% and EOD Group 2: 38.1%), followed by the 55–59 age group (34.8% and 34.4% in EOD Groups 1 and 2, respectively). Regarding comorbidities at the index date, hypertension (25.8% and 16.2% in EOD Groups 1 and 2, respectively) and depression (20.4% and 22.2% in EOD Groups 1 and 2, respectively) were the most prevalent comorbidities. Amongst participants, having one comorbidity was the most common (33.6% and 37.8% in EOD Groups 1 and 2, respectively), followed by two comorbidities (18.3% and 10.9% in EOD Groups 1 and 2, respectively). The number of concomitant drugs at the index date was highest in the 0–4 group (63.5% and 58.1% in EOD Groups 1 and 2, respectively), followed by the 5 or more group (36.5% and 41.9% in EOD Groups 1 and 2, respectively).

**TABLE 1:** The background characteristics of the participants at the index date (2 years follow‐up).

		Employees				Family members			
		EOD Group 1		Control Group 1		EOD Group 2		Control Group 2	
		*n*	%	*n*	%	*n*	%	*n*	%
*n*		712	—	3560	—	320	—	1600	—
Sex	Female	97	13.6	485	13.6	306	95.6	1530	95.6
Age	40–44	14	2.0	70	2.0	8	2.5	40	2.5
	45–49	47	6.6	235	6.6	14	4.4	74	4.6
	50–54	120	16.9	600	16.9	66	20.6	329	20.6
	55–59	248	34.8	1243	34.9	110	34.4	546	34.1
	60–64	283	39.7	1412	39.7	122	38.1	610	38.1
Comorbidities	Hypertension	184	25.8	920	25.8	52	16.2	260	16.2
	Diabetes	110	15.4	550	15.4	45	14.1	225	14.1
	Hyperlipidaemia	101	14.2	505	14.2	57	17.8	285	17.8
	Depression	145	20.4	725	20.4	71	22.2	355	22.2
	Cerebral infarction	61	8.6	305	8.6	27	8.4	135	8.4

Figure S1 shows the initial anti‐dementia drug prescribed in EOD Group 1. Donepezil was the most commonly prescribed drug (63.2%), followed by galantamine (14.9%). The mean duration of treatment with each anti‐dementia drug as a monotherapy was longest for donepezil (19.9 months) and galantamine (18.7 months).

**FIGURE 2:**
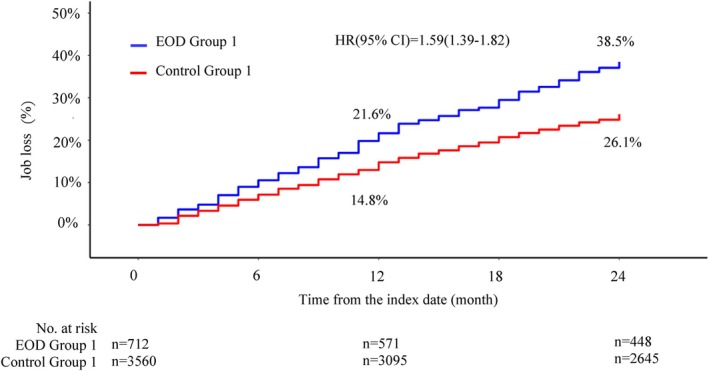
Cumulative incidence of job loss in EOD Group 1 and Control Group 1 followed up for 2 years.

**FIGURE 3:**
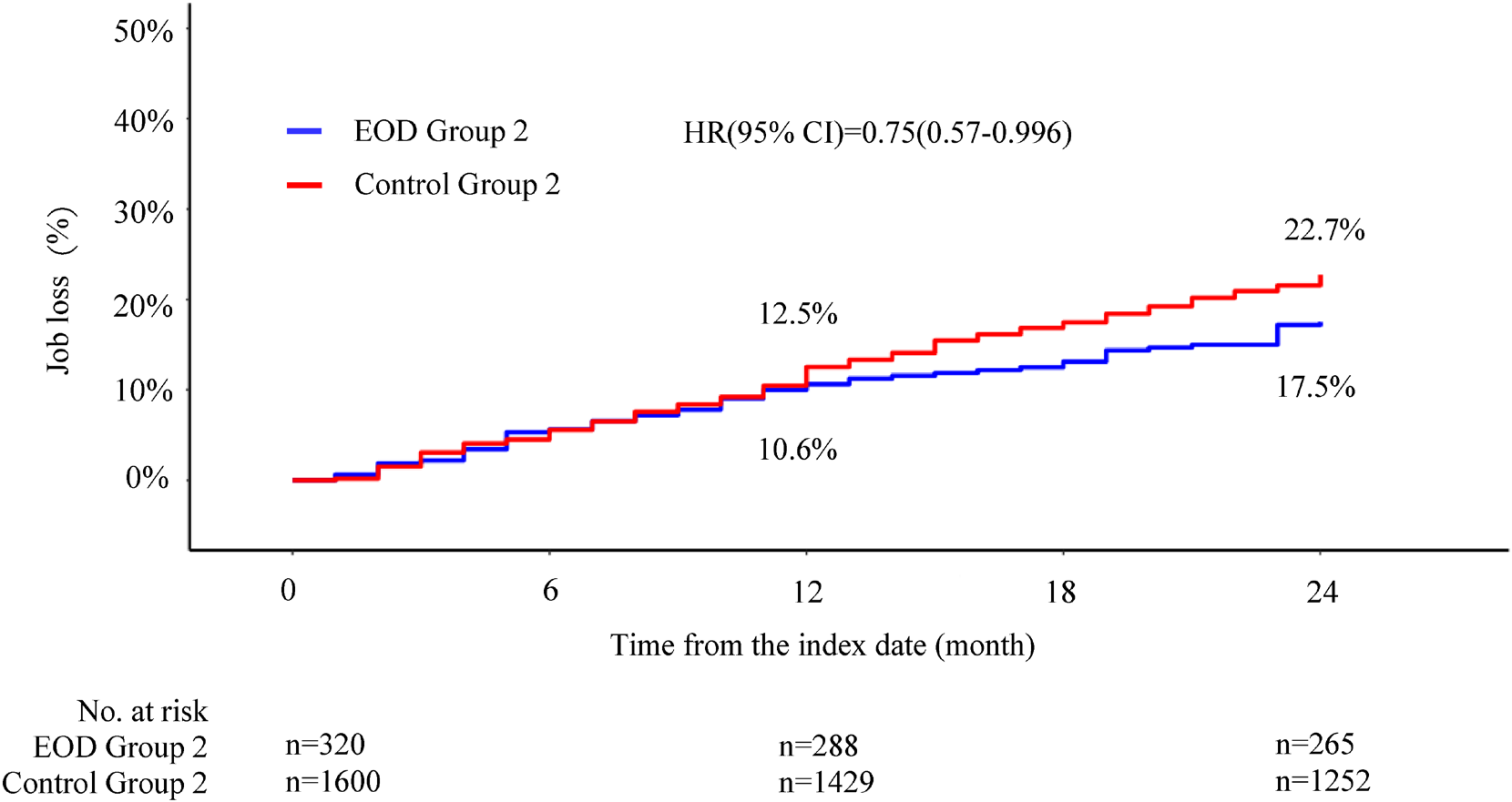
Cumulative incidence of job loss in EOD Group 2 and Control Group 2 followed up for 2 years.

The rate of job loss at 2 years was 38.5% and 26.1% in EOD Group 1 and Control Group 1, respectively (HR = 1.59, 95% CI: 1.39–1.82, *p* < 0.05) (Figure 2). Similarly, the rate was 17.5% and 22.7% in EOD Group 2 and Control Group 2 (HR = 0.75, 95% CI: 0.57–0.996, *p* = 0.046), respectively (Figure 3). The findings observed at 2 years showed a similar trend at 7 years (Figures S2 and S3). The median time to job loss was 31 and 46 months in EOD Groups 1 and 2, respectively. In addition, we analysed the rate of job loss by comorbidity in Control Group 1 at 2 years (Figure S4). The rate of job loss was highest amongst participants with concomitant cerebral infarction (39.1%), followed by those with depression (31.3%), diabetes (31.0%) and hypertension (30.1%). The target age range of this study was 40 to 64 years; however, for comparison with the previous report, we additionally performed a subgroup analysis for the participants aged 40 to 59 years. The job loss rates for EOD Group 1 and EOD Group 2 with this age range are shown in Figures S5 and S6.

Table 2 shows the background factors of participants in EOD Group 1 affecting the cumulative incidence rates of job loss at 2 years. In addition to sex, age and comorbidities, the timing of the index date was included in the model as a background factor to evaluate whether the time of diagnosis affected the rate of job loss. A multivariate Cox PHs model was used to estimate adjusted HRs, identifying several significant factors associated with job loss: sex (female) (adjusted HR = 2.18, 95% CI:1.60–2.96, *p* < 0.05), age (adjusted HR = 1.07, 95% CI: 1.04–1.10, *p* < 0.05) and hypertension (adjusted HR = 1.35, 95% CI: 1.02–1.77, *p* < 0.05).

**TABLE 2:** The background factors of participants in EOD Group 1 affecting the cumulative incidence rates of job loss at 2 years.

Participant background	Unadjusted hazard ratios from univariate Cox proportional hazards model	Adjusted hazard ratios from multivariate Cox proportional hazards model
Sex (female)	1.95 (1.45–2.63)*	2.18 (1.60–2.96)*
Age	1.07 (1.04–1.10)*	1.07 (1.04–1.10)*
Timing of the index date	1.05 (0.995–1.10)	1.03 (0.98–1.09)
Hypertension	1.38 (1.06–1.78)*	1.35 (1.02–1.77)*
Diabetes	1.11 (0.81–1.53)	1.03 (0.73–1.45)
Hyperlipidaemia	0.98 (0.70–1.37)	0.87 (0.61–1.26)
Depression	0.85 (0.63–1.15)	0.92 (0.67–1.27)
Cerebral infarction	1.46 (0.998–2.13)	1.19 (0.81–1.76)

* *p* < 0.05, statistically significant.

## 4 Discussion

In this study, we analysed JMDC data spanning 10 years and 8 months, from 1 April 2013, to 30 November 2023, which included approximately 5 million primary insured individuals aged 40–64 years. Amongst them, 712 individuals were identified as having EOD, corresponding to a rate of 14.27 per 100 000 population. This figure is lower than the point prevalence reported in a nationwide survey [3], which was 50.9 per 100 000. The discrepancy may be attributed to differences in study design and target populations. While the present study utilised a claims database of corporate health insurance enrolees—a relatively healthy and employment‐stable population—the previous report conducted a questionnaire‐based survey through health‐care facilities, long‐term care insurance facilities, disability care facilities and counselling centres, likely capturing a broader and more clinically severe patient population. Furthermore, our study applied multiple inclusion criteria: cases were required to have both a diagnosis of dementia and a prescription record for anti‐dementia medication, to be continuously enrolled in the insurance system for at least 365 days prior to the index date, and to have at least 2 years of follow‐up data available. In contrast, the nationwide survey reported the point prevalence of EOD, representing the total number of patients with dementia at a given time. Therefore, differences in the nature of the indicators used may also have contributed to the observed variation in prevalence estimates. Considering these factors, the EOD incidence rate in this study represents an estimate under specific conditions. Future research should integrate both point prevalence and cumulative incidence for a more comprehensive evaluation.

In this study, we included only participants with EOD and who were prescribed anti‐dementia drugs. In contrast, a previous report [9] included participants who either had EOD or a prescription of anti‐dementia drugs. Consequently, this study employed more rigorous criteria for participant selection than those used in the previous report. In this study, the participants were aged 40–64 years. The target age range in the previous report was 40–59 years; however, we extended the upper age limit to 64 years to enable a more comprehensive examination of the reality of EOD compared to the previous report. We believe this extension in age range allowed for a more accurate investigation of job loss. In the previous study, some participants were diagnosed with dementia but were not prescribed anti‐dementia medications. A diagnosis without a prescription may reflect either milder cases or diagnostic uncertainty. Therefore, the earlier study may have included milder cases than the present study, potentially leading to an underestimation of job loss. The rate of job loss at 12 months was higher in this study (21.6%) than in the previous report (14.0%) because of not only the increase in the target age of the participants but also differences in case definition described above. However, the difference from the control group was similar to the previous report. In this study, when focusing on cases aged 40–59—the same target age group as in the previous report—the job loss rate at 12 months in EOD Group 1 was 17.9%, which was higher than that reported previously but lower than the job loss rate for the 40–64 age group in this study. A similar trend was observed in EOD Group 2, with job loss rates of 7.8% in the previous report, 9.1% in this study's 40–59 age group and 10.6% in this study's 40–64 age group. This study applied more rigorous criteria for selecting EOD cases compared to the previous report, which may have resulted in a higher rate than the earlier findings. Although a law promoting the extension of the retirement age to 65 was enacted around 2013 in Japan, many companies still maintain a retirement age of 60, followed by 1‐year contract‐based reemployment. Therefore, the inclusion of those aged 60–64 in this study's 40–64 age group may have contributed to the higher job loss rate observed.

In this study, long‐term observation was conducted for up to 7 years. Similar to the previous study, EOD Group 1 had a higher rate of job loss than Control Group 1 at 2 years (Figure 2). The trends in the rate of job loss were similar for the 2‐ and 7‐year follow‐up periods, and the difference between EOD Group 1 and Control Group 1 was greater at 7 years than at 2 years (Figure S2). The rate of job loss at 2 years by comorbidity at EOD onset for Control Group 1 was 39.1% for cerebral infarction, 31.3% for depression, 31.0% for diabetes mellitus and 30.1% for hypertension (Figure S4). The rate of job loss at 2 years for participants with EOD was 38.5%, which was lower than that for cerebral infarction and higher than that for other comorbidities.

Conversely, EOD Group 2 had a lower rate of job loss than Control Group 2 (Figure 3). In the previous report, the rate of job loss was similar to that in the control group; however, the trend differed in this study. This change may be influenced by several factors. First, the previous report included data from April 2013 to June 2015, whereas this study utilised a longer and more recent data period, extending from April 2013 to November 2023. During this expanded timeframe, Japan implemented several dementia‐related policies, including the New Orange Plan in 2015, the National Framework for Promotion of Dementia Policies in 2019 and the Basic Act on Dementia to Promote an Inclusive Society in 2023 [28]. These policies have improved caregiver support and the availability of care services and may have made the insured individuals more comfortable with continuing their employment, even if their spouses had dementia. These factors may have contributed to the lower job loss rate observed in EOD Group 2 compared to Control Group 2; further research is needed to better understand this trend and its underlying causes. In contrast, EOD is a disease that typically affects individuals in their prime working years, and its onset is likely to lead to a significant reduction in household income [4, 5]. Moreover, there may be additional financial burdens, such as educational costs for children who are not employed, which go beyond basic living expenses. Therefore, even if the spouse of the primary insured individual develops dementia, the financial necessity of maintaining the household income may have created a situation where the primary insured individual had no choice but to continue working. The trends in the rates of job loss were similar for the 2‐ and 7‐year periods, and the difference between EOD Group 2 and Control Group 2 was greater at 7 years than at 2 years (Figure S3).

In this study, several comorbidities at EOD onset, including hypertension, hyperlipidaemia, diabetes mellitus, cerebral infarction and depression, which can influence cognitive decline or the development of dementia, were included as matching factors. In contrast, the previous study utilised only sex and age as participant background factors for matching. Therefore, our study provided a more rigorous comparison by incorporating a broader set of matched participants' background factors. We also analysed the effects of various participant background factors (Cox regression) and the administration of anti‐dementia medications, which have not been addressed in previous reports. This analysis identified age, sex (female) and hypertension as significant factors associated with job loss. The rate of job loss amongst those with hypertension in Control Group 1 was lower than that for other comorbidities such as cerebral infarction (Figure S4). However, hypertension was found to be potentially significantly associated with job loss amongst comorbidities in EOD Group 1. Hypertension is widely recognised as a risk factor for the onset of dementia, and specifically, hypertension during midlife may influence the pathophysiology of EOD through cerebrovascular disorders and cognitive decline [29, 30]. Hypertension may further accelerate cognitive deterioration even after EOD onset, leading to a decline in work performance and potentially contributing to job loss. This study suggests that hypertension may affect job loss following EOD onset. However, the status of treatment for hypertension was not examined, and the underlying mechanisms of this association remain unclear; therefore, future studies should aim to clarify this aspect. Given that hypertension at EOD onset may increase the risk of job loss, interventions such as hypertension management and lifestyle modification are recommended.

This study had some limitations. First, the specific reasons for job loss were not examined, and whether retirements were planned, or job loss was due to company bankruptcy or diseases other than EOD, remains unclear. Therefore, the exact rate of job loss due to EOD may be lower. In addition, the health insurance database used in this study predominantly covers employees of large companies above a certain size. Notably, this database does not fully represent the entire Japanese population. There may be differences in healthcare access, employment conditions and morbidity rates amongst groups with different economic and occupational backgrounds, such as non‐regular workers, self‐employed individuals and employees of small and medium‐sized enterprises. Therefore, careful interpretation is required when generalising the findings of this study. Nevertheless, studies focusing on EOD remain limited, and the findings of this study provide valuable insights into this understudied area. Given that large companies are more likely to provide support for employees with health conditions, the rate of job loss may be higher if data from small and medium‐sized companies are included.

This study focused on cases where both the dementia diagnosis code based on the ICD‐10 and the prescription information for anti‐dementia medications were confirmed. The use of both diagnostic codes and medication information to define cases has been validated in studies using claims databases for other chronic diseases [31], and this approach was adopted in the present study to enhance the certainty of the findings. However, thus far, the validity of the dementia case definition using the JMDC database has not been fully established. Further research, including chart reviews and validation against clinical evaluation criteria, is needed. Additionally, in this study, comorbidities were defined solely based on ICD‐10 codes, and it is believed that combining prescription records with ICD‐10 codes could allow for a more rigorous evaluation. Future studies should consider including prescription information for a more comprehensive analysis.

Reportedly, EOD often takes longer to be diagnosed [32, 33], and this study lacked data on the duration between the onset of subjective dementia symptoms and definitive diagnosis. It is likely that the detection of dementia is accelerated in individuals living with their spouse or children. However, data on cohabitation and separate residences were unavailable and could not be examined in this study. The limited information in the database raises the possibility that unexamined confounding factors may have influenced the results. In addition, we were unable to examine the subtype of dementia. Although only data on prescription drugs were available, information on whether these drugs were taken as prescribed was not included. The diagnosis in the database may also reflect insurance names of diseases rather than definitive clinical diagnoses. Socioeconomic status and urbanisation of residence may be associated with unemployment; however, due to the lack of relevant information in our dataset, this relationship could not be examined in the present study. In this study, only cases that had been continuously enrolled in the health insurance system for at least 365 days prior to the index date were included, allowing for sufficient baseline information and verification of healthcare utilisation prior to disease onset. This criterion is widely used in claims database studies as a standard method to ensure the reliability of diagnoses and prevent misclassification [10]. In contrast, by restricting the analysis to cases that had been continuously enrolled for at least 365 days, a relatively healthy population with stable employment and insurance coverage may have been selected. Consequently, the exclusion of short‐term enrolees and individuals with unstable employment may have led to an underestimation of the actual job loss rate. However, despite these limitations, this study provides valuable insights into job loss amongst individuals affected by EOD.

The following points, which were not fully addressed in this study, require further investigation. Since dementia is a long‐term condition, future research incorporating large cohorts and extended follow‐up periods is essential for deeper insights. Recently, disease‐modifying therapy has been introduced as a new treatment for Alzheimer's disease; however, this study covered the period before the launch of these medicines.

Japan is one of the world's rapidly ageing countries, facing a significant challenge due to its declining birth rate. Therefore, the middle‐aged and older populations are increasingly expected to take on active roles within the workforce, and it is essential to explore ways to create supportive work environments. Conducting research to develop workplaces in which individuals with mild cognitive impairment and mild dementia can work for as long as possible may be a significant step towards addressing this societal challenge.

## 5 Conclusion

Participants with EOD had a higher rate of job loss than those in the control group. This study also examined participant background factors associated with job loss amongst participants with EOD, suggesting that managing comorbidities at EOD onset and implementing lifestyle modifications may be beneficial. Notably, the rate of job loss was lower amongst insured participants' spouses with EOD compared to the control group.

EOD presents significant socioeconomic challenges for patients, their families and society. Patients with EOD may be employed or have financially dependent children, and the company may have to consider hiring replacements. Therefore, examining the time to job loss after the EOD onset is crucial for predicting its economic impact and considering effective support strategies. This study provides valuable insights for reducing the rate of job loss by enforcing workplace health promotion for employees with EOD, especially women and those with hypertension.

## Funding

This study was supported by Eisai Co. Ltd.

## Consent

All information was anonymized by JMDC Inc., and informed consent was not required from the individuals included in this study.

## Conflicts of Interest

Authors K.S. and Y.H. are employees of Eisai Co. Ltd. The other authors declare no conflicts of interest.

## Supporting information


**Figure S1:** The first anti‐dementia drug administered in EOD Group 1.

**Figure S2:** Cumulative incidence of job loss in EOD Group 1 and Control Group 1 followed up for 7 years.

**Figure S3:** Cumulative incidence of job loss in EOD Group 2 and Control Group 2 followed up for 7 years.

**Figure S4:** Cumulative incidence of job loss per comorbidity in the Control Group 1 followed up for 2 years.

**Figure S5:** Cumulative incidence of job loss in EOD Group 1 and Control Group 1 followed up for 2 years (40–59 years).

**Figure S6:** Cumulative incidence of job loss in EOD Group 2 and Control Group 2 followed up for 2 years (40–59 years).

**Table S1:** Comorbidities defined based on ICD‐10 criteria.

**Table S2:** The background of the primary insured participants at the index date (7 years follow‐up).

## Data Availability

The data are available for purchase from JMDC Inc. Restrictions apply to the availability of the data used in this study because of contractual agreements between JMDC Inc. and the health insurance associations. For inquiries regarding the accessibility of the dataset, please contact JMDC Inc. (https://www.jmdc.co.jp/en/).
