# Supplementary material for: Effect of Early‐Onset Dementia on Job Loss in Japan: A Matched Cohort Database Study Using Health Insurance Claims Data
Source: Psychogeriatrics. 2025 Nov 28;26(1):e70117. doi: 10.1111/psyg.70117 (PMC12661630; doi:10.1111/psyg.70117)
Supplement: Supplementary file 1 — Figure S1: The first anti‐dementia drug administered in EOD Group 1. [file PSYG-26-0-s005.docx]

Others（Combination of two drugs, etc.）

1.0%（7/712）


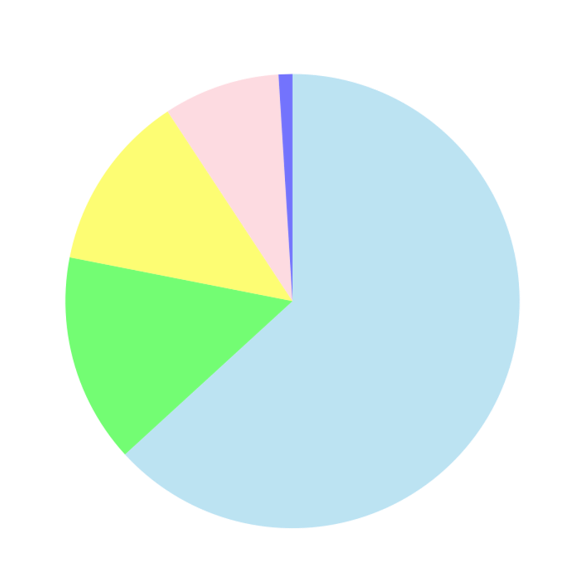


Rivastigmine

8.3%（59/712）

Memantine

12.6%（90/712）

Donepezil

63.2%（450/712）

Galantamine

14.9%（106/712）

Supplementary Figure 1 The first anti-dementia drug administered in EOD Group 1
