# Supplementary material for: Effect of Early‐Onset Dementia on Job Loss in Japan: A Matched Cohort Database Study Using Health Insurance Claims Data
Source: Psychogeriatrics. 2025 Nov 28;26(1):e70117. doi: 10.1111/psyg.70117 (PMC12661630; doi:10.1111/psyg.70117)
Supplement: Supplementary file 2 — Figure S2: Cumulative incidence of job loss in EOD Group 1 and Control Group 1 followed up for 7 years. [file PSYG-26-0-s001.docx]

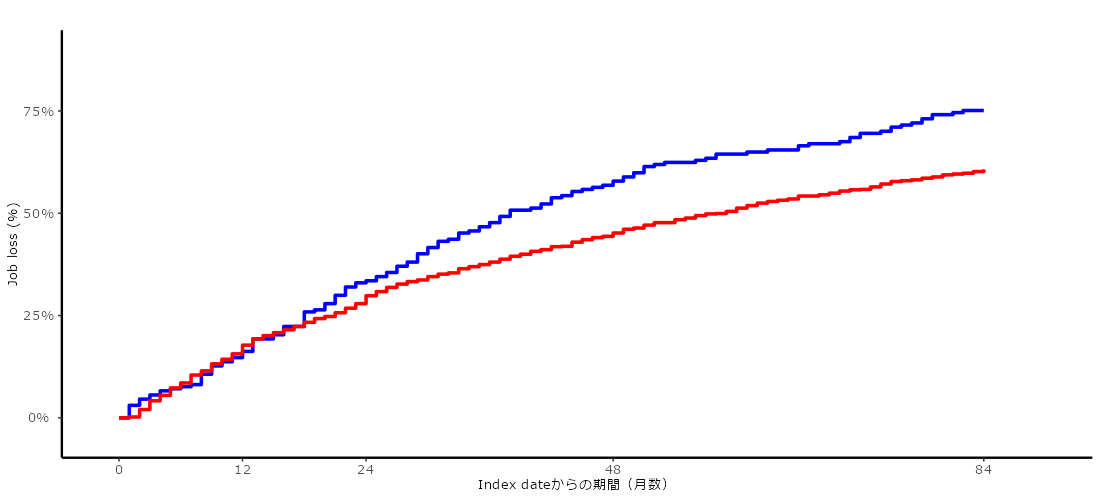


Job loss（%）

57.9%

45.2%

0%

25%

50%

75%

12

0

48

84

24

75.1%

EOD Group 1

Control Group 1

60.7%

HR(95% CI)=1.41(1.17-1.68)

Time from the index date (month)

No. at risk

EOD Group 1

Control Group 1

n=85

n=548

n=50

n=392

n=197

n=985

Supplementary Figure 2 Cumulative incidence of job loss in EOD Group 1 and Control Group 1 followed up for 7 years
