# Supplementary material for: Effect of Early‐Onset Dementia on Job Loss in Japan: A Matched Cohort Database Study Using Health Insurance Claims Data
Source: Psychogeriatrics. 2025 Nov 28;26(1):e70117. doi: 10.1111/psyg.70117 (PMC12661630; doi:10.1111/psyg.70117)
Supplement: Supplementary file 3 — Figure S3: Cumulative incidence of job loss in EOD Group 2 and Control Group 2 followed up for 7 years. [file PSYG-26-0-s007.docx]

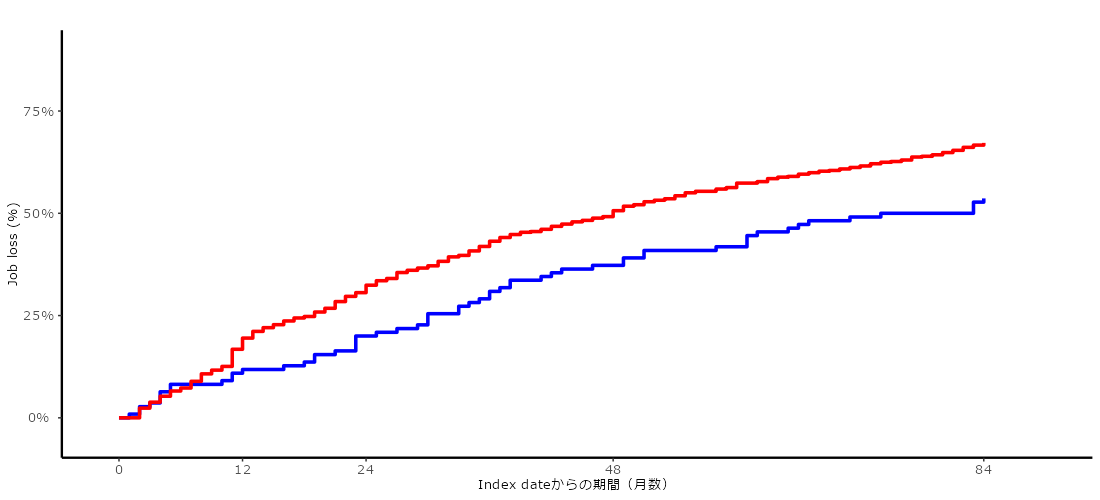


HR(95% CI)=0.68(0.51-0.89)

37.3%

50.6%

0%

75%

Job loss（%）

0

24

48

84

50%

12

25%

67.2%

EOD Group 2

Control Group 2

53.6%

No. at risk

EOD Group 2

Control Group 2

Time from the index date (month)

n=70

n=279

n=52

n=183

n=110

n=550

Supplementary Figure 3 Cumulative incidence of job loss in EOD Group 2 and Control Group 2 followed up for 7 years
