# Supplementary material for: Effect of Early‐Onset Dementia on Job Loss in Japan: A Matched Cohort Database Study Using Health Insurance Claims Data
Source: Psychogeriatrics. 2025 Nov 28;26(1):e70117. doi: 10.1111/psyg.70117 (PMC12661630; doi:10.1111/psyg.70117)
Supplement: Supplementary file 4 — Figure S4: Cumulative incidence of job loss per comorbidity in the Control Group 1 followed up for 2 years. [file PSYG-26-0-s003.docx]

Cerebral infarction 39.1%


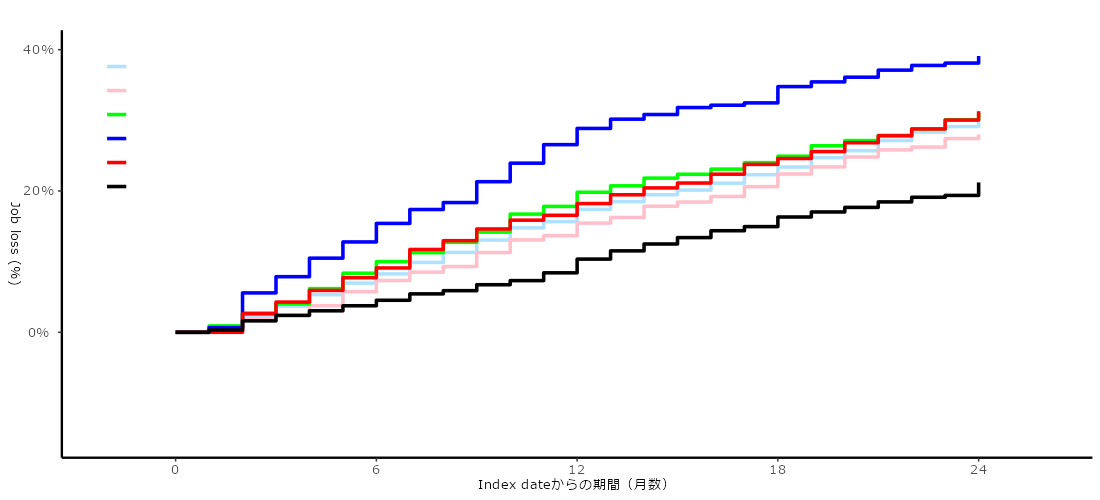


Depression 31.3%

40%

Diabetes 31.0%

Hypertension 30.1%

Job loss（%）

Hyperlipidaemia 28.0%

20%

No comorbidities 21.2%

0%

Cerebral infarction

Depression

Diabetes

Hyperlipidemia

No comorbidities

Hypertension

6

0

12

18

24

Time from the index date (month)

No. at risk

Cerebral infarction

Depression

Diabetes

Hypertension

Hyperlipidaemia

No comorbidities

n=305

n=725

n=550

n=920

n=505

n=1545

n=186

n=501

n=379

n=643

n=362

n=1230

Supplementary Figure 4 Cumulative incidence of job loss per comorbidity in the Control Group 1 followed up for 2 years
