# Supplementary material for: Effect of Early‐Onset Dementia on Job Loss in Japan: A Matched Cohort Database Study Using Health Insurance Claims Data
Source: Psychogeriatrics. 2025 Nov 28;26(1):e70117. doi: 10.1111/psyg.70117 (PMC12661630; doi:10.1111/psyg.70117)
Supplement: Supplementary file 5 — Figure S5: Cumulative incidence of job loss in EOD Group 1 and Control Group 1 followed up for 2 years (40–59 years). [file PSYG-26-0-s004.docx]

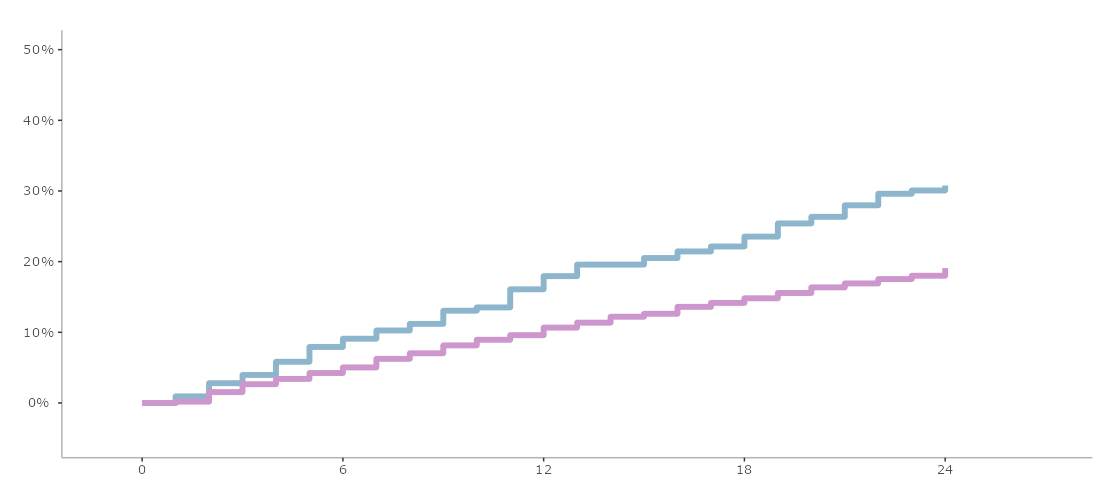


50%

EOD Group 1

HR(95% CI)=1.73(1.43-2.11)

Control Group 1

40%

Job loss（%）

30.8%

30%

17.9%

20%

19.1%

10%

10.7%

0%

24

18

12

0

6

Time from the index date (month)

n=300

n=1740

n=360

n=1940

No. at risk

EOD Group 1

Control Group 1

n=429

n=2148

Supplementary Figure 5 Cumulative incidence of job loss in EOD Group 1 and Control Group 1 followed up for 2 years (40 to 59 years)
