# Supplementary material for: Effect of Early‐Onset Dementia on Job Loss in Japan: A Matched Cohort Database Study Using Health Insurance Claims Data
Source: Psychogeriatrics. 2025 Nov 28;26(1):e70117. doi: 10.1111/psyg.70117 (PMC12661630; doi:10.1111/psyg.70117)
Supplement: Supplementary file 6 — Figure S6: Cumulative incidence of job loss in EOD Group 2 and Control Group 2 followed up for 2 years (40–59 years). [file PSYG-26-0-s002.docx]

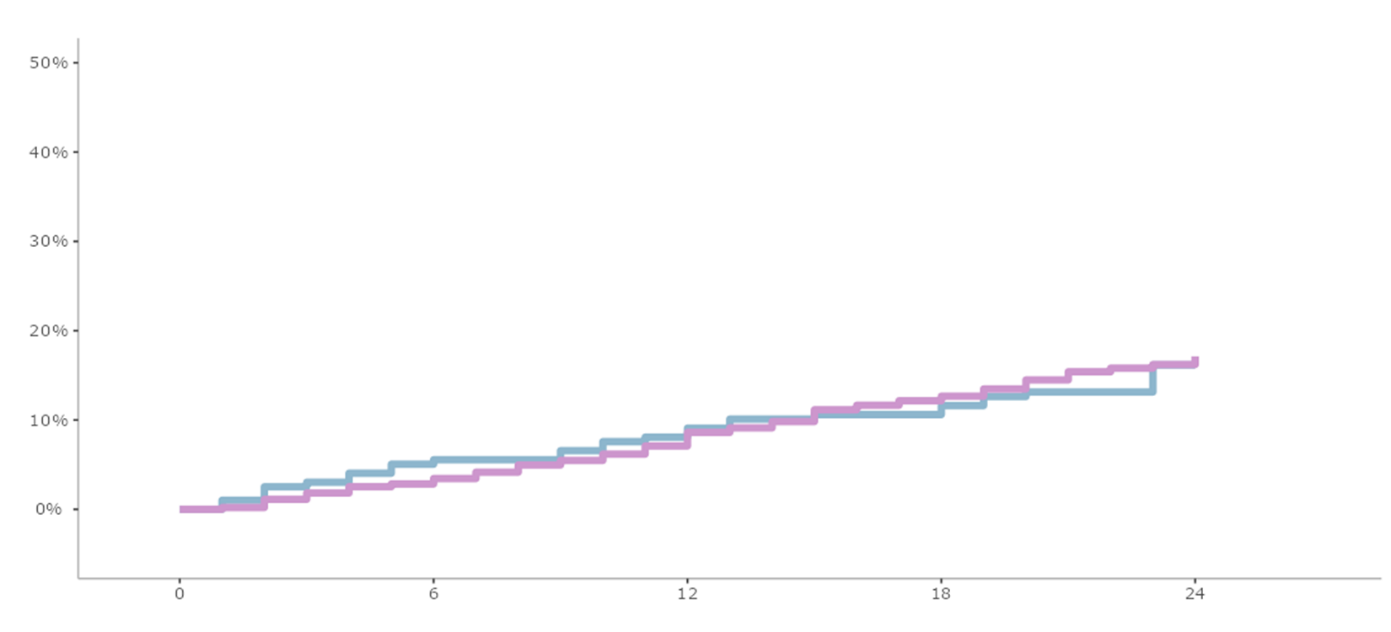


50%

EOD Group 2

HR(95% CI)=0.95(0.65-1.38)

Control Group 2

40%

Job loss（%）

30%

17.1%

20%

9.1%

10%

16.2%

8.6%

0%

n=198

n=989

n=172

n=827

n=182

n=917

No. at risk

EOD Group 2

Control Group 2

Time from the index date (month)

24

18

12

0

6

Supplementary Figure 6 Cumulative incidence of job loss in EOD Group 2 and Control Group 2 followed up for 2 years (40 to 59 years)
