# Supplementary material for: Effect of Early‐Onset Dementia on Job Loss in Japan: A Matched Cohort Database Study Using Health Insurance Claims Data
Source: Psychogeriatrics. 2025 Nov 28;26(1):e70117. doi: 10.1111/psyg.70117 (PMC12661630; doi:10.1111/psyg.70117)
Supplement: Supplementary file 7 — Table S1: Comorbidities defined based on ICD‐10 criteria. [file PSYG-26-0-s008.docx]

Supplementary Table 1 Comorbidities defined based on ICD-10 criteria

| Comorbidities | ICD-10 |
| --- | --- |
| Hypertension | I10, I11, I12, I13, I14, I15 |
| Diabetes | E10, E11, E12, E13, E14, Insulin receptor disorder (standardized disease name), Lipoatrophic diabetes (standardized disease name) |
| Hyperlipidaemia | E784, E785 |
| Depression | F32, F33 |
| Cerebral infarction | I630, I631, I632, I633, I634, I635, I636, I638, I639 |
