# Supplementary material for: Effect of Early‐Onset Dementia on Job Loss in Japan: A Matched Cohort Database Study Using Health Insurance Claims Data
Source: Psychogeriatrics. 2025 Nov 28;26(1):e70117. doi: 10.1111/psyg.70117 (PMC12661630; doi:10.1111/psyg.70117)
Supplement: Supplementary file 8 — Table S2: The background of the primary insured participants at the index date (7 years follow‐up). [file PSYG-26-0-s006.docx]

Supplementary Table 2 The background of the primary insured participants at the index date　　　 (7 years follow-up)

|  |  | Employees | | | | Family members | | | |
| --- | --- | --- | --- | --- | --- | --- | --- | --- | --- |
|  |  | EOD Group 1 | | Control Group 1 | | EOD Group 2 | | Control Group 2 | |
|  |  | n | % | n | % | n | % | n | % |
| n |  | 197 | - | 985 | - | 110 | - | 550 | - |
| Sex | Female | 21 | 10.7 | 105 | 10.7 | 105 | 95.5 | 525 | 95.5 |
| Age | 40 to 44 | 4 | 2.0 | 20 | 2.0 | 4 | 3.6 | 20 | 3.6 |
|  | 45 to 49 | 15 | 7.6 | 75 | 7.6 | 4 | 3.6 | 23 | 4.2 |
|  | 50 to 54 | 35 | 17.8 | 175 | 17.8 | 22 | 20.0 | 111 | 20.2 |
|  | 55 to 59 | 64 | 32.5 | 319 | 32.4 | 40 | 36.4 | 194 | 35.3 |
|  | 60 to 64 | 79 | 40.1 | 396 | 40.2 | 40 | 36.4 | 202 | 36.7 |
| Comorbidities | hypertension | 44 | 22.3 | 220 | 22.3 | 14 | 12.7 | 70 | 12.7 |
|  | diabetes | 35 | 17.8 | 175 | 17.8 | 19 | 17.3 | 95 | 17.3 |
|  | hyperlipidemia | 23 | 11.7 | 115 | 11.7 | 18 | 16.4 | 90 | 16.4 |
|  | depression | 40 | 20.3 | 200 | 20.3 | 26 | 23.6 | 130 | 23.6 |
|  | cerebral infarction | 18 | 9.1 | 90 | 9.1 | 8 | 7.3 | 40 | 7.3 |
